# Osteoblast precursors and inflammatory cells arrive simultaneously to sites of a trabecular-bone injury

**DOI:** 10.1080/17453674.2018.1481682

**Published:** 2018-06-05

**Authors:** Magnus Bernhardsson, Per Aspenberg

**Affiliations:** Orthopedics, Department of Clinical and Experimental Medicine, Faculty of Health Sciences, Linköping University, Linköping, Sweden.

## Abstract

Background and purpose — Fracture healing in the shaft is usually described as a sequence of events, starting with inflammation, which triggers mesenchymal tissue formation in successive steps. Most clinical fractures engage cancellous bone. We here describe fracture healing in cancellous bone, focusing on the timing of inflammatory and mesenchymal cell type appearance at the site of injury

Material and methods — Rats received a proximal tibial drill hole. A subgroup received clodronate-containing liposomes before or after surgery. The tibiae were analyzed with micro-CT and immunohistochemistry 1 to 7 days after injury.

Results — Granulocytes (myeloperoxidase) appeared in moderate numbers within the hole at day 1 and then gradually disappeared. Macrophage expression (CD68) was seen on day 1, increased until day 3, and then decreased. Mesenchymal cells (vimentin) had already accumulated in the periphery of the hole on day 1. Mesenchymal cells dominated in the entire lesion on day 3, now producing extracellular matrix. A modest number of preosteoblasts (RUNX2) were seen on day 1 and peaked on day 4. Osteoid was seen on day 4 in the traumatized region, with a distinct border to the uninjured surrounding marrow. Clodronate liposomes given before the injury reduced the volume of bone formation at day 7, but no reduction in macrophage numbers could be detected.

Interpretation — The typical sequence of events in shaft fractures was not seen. Mesenchymal cells appeared simultaneously with granulocyte and macrophage arrival. Clodronate liposomes, known to reduce macrophage numbers, seemed to be associated with the delineation of the volume of tissue to be replaced by bone. Most fracture healing studies in animal models concern cortical bone in shafts. However, most fractures in patients occur in cancellous bone in the metaphysis, such as the distal radius or in the vertebrae. A growing body of evidence suggests that there are important differences between the healing processes in cortical and cancellous bone.

Shaft fracture healing relies on the recruitment of cells from external sources, such as the periosteum, surrounding soft tissues and blood circulation, and proceeds by forming a large, expanding callus (Kumagai et al. 2008). In contrast, the healing of cancellous bone, which is rich in mesenchymal stem cells, is strictly localized within the confinements of the injured region (Bernhardsson et al. 2015). John Charnley observed this phenomenon during the 1950s when working with knee arthrodeses. The cancellous bone formation in the traumatized regions rarely extended more than a couple of millimeters, meaning that just a small gap between Charnley’s resection surfaces could jeopardize healing (Charnley and Baker 1952).

The complex interactions between bone and immune cells are incompletely understood, but macrophages seem to play a major role in bone healing. The depletion of macrophages with clodronate liposomes impairs the healing of cancellous bone, both mechanically and morphometrically (Sandberg et al. 2017).

With this descriptive study we explored the timing of cell arrival at the site of cancellous bone injury and the effect of attempted monocyte depletion, to get an idea of the possible interaction between inflammation and new bone formation. 

## Material and methods

### Experimental overview

2 experiments were conducted for this paper. The first monitored the healing of drill holes in cancellous bone during the first 5 days. 30 male SOPF Sprague-Dawley rats, 10 weeks old (Janvier, Saint-Berthevin Cedex, France), weighing 444 g (SD 23) received bilateral drill holes in their proximal tibiae. The rats were randomized to be killed either 1, 2, 3, 4, or 5 days after surgery, 6 animals each day. The tibiae were harvested and prepared for histology and immunohistochemistry.

The second experiment monitored the effect of macrophage depletion in healing of drill holes in cancellous bone. 36 male SOPF Sprague-Dawley rats, 10 weeks old (Janvier, Saint-Berthevin Cedex, France), weighing 503 g (SD 25) received a drill hole in their right proximal tibiae. 24 of the animals received a single injection in the tail vein of 20 mg/kg of clodronate liposomes (5 mg/mL; Liposoma BV, Amsterdam, Netherlands) either 24 hours prior to surgery or 24 hours after surgery. The animals were killed on day 3 or 7 after surgery (n = 6). The tibiae were harvested, analyzed with micro-CT and prepared for histology and immunohistochemistry.

### Surgical procedure

The surgery was performed under isoflurane anesthesia and aseptic conditions. A 1.2 mm drill hole was made by hand, using a 18G syringe needle, about 4 mm below the growth plate in the antero-medial surface of the proximal tibia. The skin was then sutured, and analgesia was given every 8–12 hours for the following 48 hours. This procedure has been described in greater detail before (Bernhardsson et al. 2015).

### Micro-CT

The clodronate-treated tibiae were analyzed with micro-CT (Skyscan 1174, v. 2; Bruker, Aarteselaar, Belgium). In a 180° scan, a pixel size of 11.2 µm, aluminum filter 0.5 mm, rotation step of 0.4° and frame averaging of 3, and energy settings of 50 kV and 800 µA were used to acquire radiographic images. NRecon (Skyscan, v. 1.6.8.0; Aarteselaar, Belgium) was used to reconstruct the images and correct them for ring artifacts and beam hardening. Within the former drill holes, a volume of interest (VOI) was defined as a cylinder with a diameter of 1.2 mm and 1.5 mm in length into the bone marrow cavity, starting from the endosteal side. 2 hydroxyapatite standards of known density (0.25 and 0.75 g/cm3) were used to calibrate the bone mineral density. The total bone volume (BV/TV) of the VOIs was analyzed in CTAn (Skyscan, v. 1.10; Aarteselaar).

### Histology

The tibiae were fixed in 4% paraformaldehyde for 24 hours before they were put in 10% EDTA for 10 days for decalcification. The demineralized tibiae were dehydrated in a series of increasing concentration of ethanol and embedded in paraffin for sectioning. The tibiae were sectioned longitudinally, perpendicular to the drill holes, in 4 µm sections and stained with hematoxylin and eosin.

### Immunohistochemistry

The tibiae were prepared for immunohistochemical staining of granulocytes (myeloperoxidase, MPO), macrophages (CD68), mesenchymal cells (vimentin) and preosteoblasts (runt-related transcription factor 2, RUNX2).

The paraffin embedded tibiae were sectioned longitudinally in 4 µm sections. Care was taken to make the sections perpendicular to the direction of the drill hole. Sections were dewaxed and rehydrated in a series of decreasing concentration of ethanol. Heat mediated antigen retrieval was performed by incubating the slides in Tris-EDTA buffer, pH 9, for 60 min. Blocking of endogenous peroxidase in 3% hydrogen peroxide, permeabilization with 0.25% Triton X-100 and blocking with Protein Block, Serum-free (X0909, DAKO, Santa Clara, CA, USA) was performed before the sections where incubated with primary antibodies for 80 min (overnight for RUNX2) in room temperature. The following antibodies were used: anti-MPO (1:200, ab9535, Abcam, Cambridge, UK), anti-CD68 (1:250, ab125212, Abcam, Cambridge, UK), anti-vimentin (1:1000, ab92547, Abcam, Cambridge, UK), and anti-RUNX2 (1:100, ab23981, Abcam, Cambridge, UK). The sections were rinsed in TBS and then incubated with biotin-labeled goat anti-rabbit secondary antibody (1:200, E0432, DAKO, Santa Clara, CA, USA). After rinsing, the sections were incubated with Vectastain Elite ABC Kit (PK-6100, Vector Laboratories, Burlingame, CA, USA) according to the manufacturer’s instructions. Staining was visualized with Vector® VIP Peroxidase Substrate Kit (SK-4600, Vector Laboratories, Burlingame, CA, USA) and counterstained with methyl green.

The quantification of stained MPO, CD68, vimentin, and RUNX2 positive cells was conducted using a light microscope, equipped with a color camera. Images were taken with 12.5X magnification, 1 image inside and 1 outside of the traumatized region for each specimen. The aim was to pick sites that were homogenous, without any trabeculae or artifacts interfering. The examiner was blinded regarding time point. Positively stained cells were quantified in the images using image analysis software (ImageJ; https://imagej.nih.gov/ij/index.html).

## Statistics

This was a descriptive study, and no hypothesis was specified in advance. We therefore refrained from formulating and testing hypotheses in retrospect, and thus present 95% confidence intervals (CI) for the differences between group means, based on t-distributions, but no p-values.

## Ethics, funding, and potential conflicts of interest

All procedures were approved by the Research Ethics Board in Linköping, Sweden, in accordance with the Swedish Animal Welfare Act (1988:534) and EU-Directive 2010/63/EU. The registration ID is 49-15. This study was supported by the Swedish Research Council (2031-47-5), AFA insurance company, EU 159 7th framework program (FP7/2007-2013, grant 279239) and a specific grant from Linköping 160 University. No conflicts of interest were declared.

## Results

### Exclusions

2 animals in the –24 h clodronate liposome group died postoperatively for unknown reasons.

## Cell arrival in the drill hole

**Day 1** — The drill hole was easily detected as a circular area with mainly necrotic material, erythrocytes, and scattered cells. Granulocytes (myeloperoxidase, MPO) and macrophages (CD68) were present in moderate numbers. Mesenchymal cells (vimentin-positive cells with a morphology spanning from spindle-shaped cells to osteocytes) and preosteoblasts (RUNX2; mainly a subcategory of vimentin-labeled cells) were all present in moderate numbers. Mesenchymal cells, mostly with a spindle-shaped morphology, were particularly present in the periphery of the lesion, while some cells with round morphology could be seen in the center.

**Figure 1.**
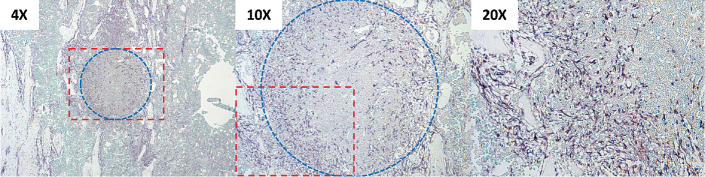
Immunohistochemistry image (vimentin) of drill hole, 2 days after trauma. Spindle-shaped mesenchymal cells could be seen in the periphery of the lesion (blue marking), forming a circle at the interface.

**Day 2** — Granulocytes had decreased in numbers, while macrophages showed a slight increase. Mesenchymal cells and preosteoblasts (a minority of the mesenchymal cells) both showed more than a 2-fold increase from day 1, and now had infiltrated further into the lesion. The mesenchymal cells in the periphery of the lesion now appeared as a circle around it (Figure 1).

**Day 3** — Granulocytes kept on decreasing. Macrophages had increased in numbers 3-fold compared with day 2. Mesenchymal cells could now be seen in the whole traumatized region, still with mainly spindle-shaped morphology, but now producing extracellular matrix. Many of them were also labeled as preosteoblasts, which were seen in the entire lesion and increased in numbers.

**Day 4** — Granulocytes were now very few, and macrophages had decreased in numbers. Most mesenchymal cells were preosteoblasts. They had increased in numbers and now occupied the whole region. Formation of osteoid tissue could now be seen within the borders of the traumatized region.

**Figure 2.**
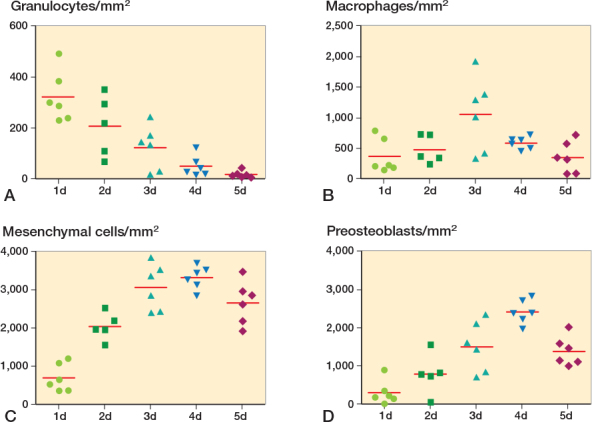
Quantification of cell populations in drill holes in proximal tibia. Granulocytes (A; myeloperoxidase, MPO). Macrophages (B; CD68). Mesenchymal cells (C; vimentin). Preosteoblasts (D; RUNX2).

**Day 5** — Granulocytes were almost absent. Macrophages were few. Vimentin-positive cells with a more osteoblastic (cuboidal) or osteocytic (round) morphology could now be seen in the region. Many of these cells were no longer RUNX2-positive (Figure 2).

## Outside the drill hole

No greater alteration in numbers of cells could be seen over time in the intact marrow, outside of the traumatized region, for any of the cell populations (Figure 5, see Supplementary data).

## Effect of clodronate liposome treatment

**Figure 3.**
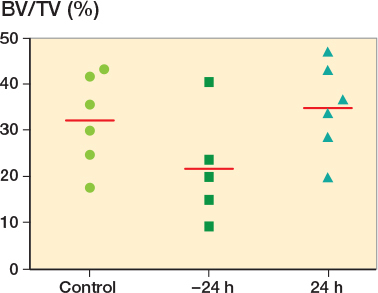
Micro-CT analysis of drill holes in proximal tibia, 7 days after trauma. Clodronate liposomes given 24 hours prior to trauma reduced the bone formation (BV/TV) by 33% (CI 14–79) compared with controls. However, this effect was absent when clodronate liposomes were administered 24 hours after trauma.

**Figure 4.**
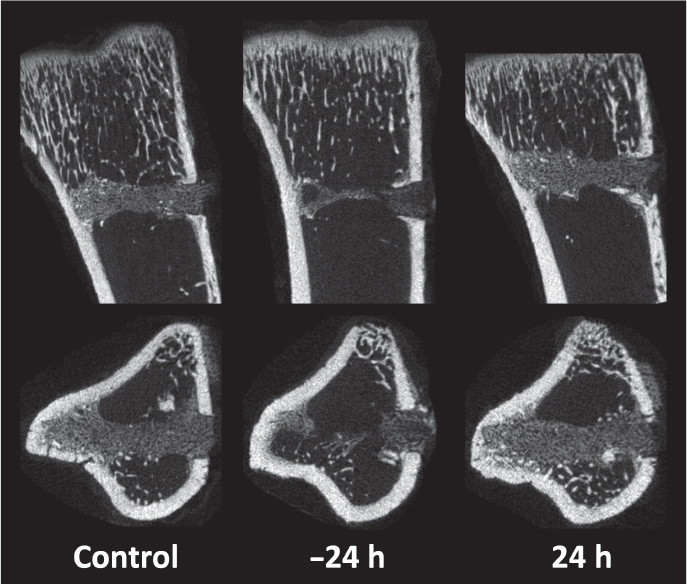
Micro-CT images of drill holes in proximal tibia, 7 days after trauma. Clodronate liposomes administered 24 hours prior to trauma impaired the bone formation in the marrow compartment, but not when given 24 hours after trauma.

**Micro-CT** — The drill holes were filled with new woven bone after 7 days in the controls. Clodronate liposomes given 24 hours before trauma reduced bone volume (BV/TV) by 33% (CI: 14 to 79) compared with controls, but clodronate liposomes administered 24 hours after trauma had no such effect (Figures 3–4).  

## Discussion

We characterized the cell composition within the spatial confinement of a cancellous bone injury. Mesenchymal cells and inflammatory cells arrived simultaneously at the injury site within the first 24 hours. Clodronate liposomes administered before trauma reduced the regenerated bone volume after 7 days.

The simultaneous arrival of mesenchymal and inflammatory cells is an important difference from the healing pattern of shaft fractures. In the literature, shaft fracture healing is described as a sequence of events with overlapping phases, where inflammatory cells are the first to arrive at the injured site before mesenchymal cells are recruited and initiate healing (Kumagai et al. 2008, Ono and Takayanagi 2017). Recruitment of mesenchymal cells from distant sources seems to be required, since mesenchymal stem cells are few at the site of a cortical shaft fracture. If the inflammatory response is attenuated, using NSAIDs, the healing of shaft fractures is impaired (Sandberg and Aspenberg 2015). There is, therefore, a need of recruitment signals from inflammatory cells that have arrived first at the injured site. However, mesenchymal cell activation is not necessarily dependent on inflammatory cells. Mesenchymal cells can respond directly to stimuli associated with injury, such as damage-associated molecular patterns (DAMPs) and mechanical stimuli (Lotfi et al. 2011, Pistoia and Raffaghello 2011, Delaine-Smith and Reilly 2012).

In contrast to the cortical shaft, cancellous bone is rich in mesenchymal cells with a stronger osteogenic potential. Therefore, a more efficient healing response can be expected in cancellous bone compared with shaft fractures (Siclari et al. 2013).

Stability of fractures is a factor worth mentioning, and since our drill hole model is of a more stable nature, could we expect different behavior if we introduced instability to some degree to the injury? We cannot see how the addition of instability in a cancellous bone injury would affect the timing of inflammatory or mesenchymal cell arrival. However, a change in cell composition and tissue formation could be expected. Probably there would be more chondrocytes initially at the site, producing cartilaginous tissue, securing a primary stable structure before bone formation (Claes et al. 2012).

The new bone in the former drill hole had the shape of a cylinder with the same radius as the hole. Clodronate liposomes led to a volume reduction in the new bone cylinder, so that the entire lesion was not replaced with bone, even though the bone that was formed looked qualitatively the same on CT and histology. We have previously seen a reduction in new-formed bone volume due to clodronate liposomes in a screw fixation model in mice, where the reduction in bone volume led to a poorer fixation of the screw and a lower pull-out force (Sandberg et al. 2017). We then speculated from our results that macrophages may have a role in inducing mesenchymal proliferation. However, similar to this study, we could not see any local effect of clodronate on either macrophages or mesenchymal cell numbers in the lesion or surrounding intact tissue, either on day 1 or on day 3 after surgery.

Prior to this study we thought that clodronate liposomes depleted macrophages generally in the circulation and tissues, including the bone marrow. However, in our case, where only 1 injection of clodronate liposomes was administered, it seems that circulating monocytes are primarily depleted (Sunderkötter et al. 2004). The liposomes cannot penetrate the vascular endothelium, and therefore a longer treatment period with repeated injections is needed for successful depletion of resident macrophages in tissues. Other evidence shows that tissue-resident macrophages are segregated from circulating monocytes and can repopulate by themselves through local proliferation (Hashimoto et al. 2013). Some subpopulations of macrophages might even increase in numbers upon monocyte depletion (Côté et al. 2013).

It is an enigma how the mesenchymal cells manage to restrict bone formation only to the drill hole, with such a sharp demarcation to the surrounding tissue. We now speculate that circulating monocytes, arriving in the hematoma from ruptured blood vessels into the traumatized region, deposit molecules in the hematoma like a “scent blueprint,” which determines the shape of the volume to be filled with new bone several days later. A disturbed “scent blueprint” might explain why monocyte depletion only at the time of trauma leads to reduced new bone volume several days later.

The hematoma is known to be important for fracture healing, and early hematoma ablation in shafts fractures leads to poor healing (Grundnes and Reikerås 1993). Our data suggest that monocytes in the hematoma are also important for osteogenesis in cancellous bone.

A limitation of this study is the lack of data on a corresponding drill hole injury in cortical bone, as a control, to compare the influx and timing of inflammatory and osteoprogenitor cells. Other limitations are the lack of data which confirms monocyte depletion, and that we have done this study in rats. Most of the earlier publications concerning monocyte/macrophage depletion have been conducted in mice, and we do not know how, or if, clodronate liposomes might have a different effect between species, even though our data correspond with earlier studies.

In summary, mesenchymal and inflammatory cells appear to be activated simultaneously upon trauma in cancellous bone. This is different from the sequential events in shaft fracture healing. Early monocyte depletion reduces late bone volume, suggesting that the presence of monocytes in the hematoma during the first days is crucial. 

## Supplementary data

Figure 5 is available as supplementary data in the online version of this article, http://dx.doi.org/.1080/17453674.2018.1481682.

Both authors were involved in designing the study, analyzing the data, preparing and approving the submitted manuscript. MB conducted the experiments and acquired the data.  

*Acta* thanks Olav Reikerås and other anonymous reviewers for help with peer review of this study.

## Supplementary Material

Supplemental Material
